# Toxic optic neuropathy in the setting of docetaxel chemotherapy: a case report

**DOI:** 10.1186/1471-2415-14-18

**Published:** 2014-02-24

**Authors:** Thomas P Moloney, Wen Xu, Kristopher Rallah-Baker, Niara Oliveira, Natasha Woodward, Jonathon J Farrah

**Affiliations:** 1 Department of Ophthalmology, The Royal Brisbane and Women’s Hospital, Herston, QLD, Australia; 2 Department of Medical Oncology, Mater Misericordiae Hospital, South Brisbane, QLD, Australia; 3 Department of Ophthalmology, Mater Misericordiae Hospital, South Brisbane, QLD, Australia

**Keywords:** Toxic optic neuropathy, Toxic, Optic neuropathy, Docetaxel, Chemotherapy

## Abstract

**Background:**

To describe the first reported case of toxic optic neuropathy secondary to docetaxel (Taxotere®) chemotherapy.

**Case presentation:**

A 53-year-old female presented with predominantly unilateral visual loss, but extensive bilateral visual field defects and bilateral optic nerve head swelling 2 weeks after first dose of docetaxel (Taxotere®) and trastuzumab (Herceptin®) chemotherapy for a left sided node-positive, HER2 positive breast cancer. Extensive investigation ruled out other causes of optic neuropathy. She was treated with high dose corticosteroids intravenously for 1 week then a tapering oral dose over 8 weeks. Visual field defects gradually resolved and visual acuity improved. Docetaxel chemotherapy was discontinued but targeted therapy with trastuzumab continued without further complication.

**Conclusion:**

Docetaxel can cause a toxic optic neuropathy possibly due to an ischemic or neurotoxic mechanism at the optic nerve head. With cessation of docetaxel therapy and treatment with systemic corticosteroids, visual recovery can occur without significant residual visual deficit.

## Background

Docetaxel is an anticancer cytotoxic that belongs to the taxane family. This drug binds to β-tubulin to inhibit the disassembly of microtubules, thus inhibiting cell replication and inducing apoptosis [1]. The taxanes are derived from the bark of the Pacific Yew *(Taxus brevfolia)*[2] and are widely accepted as evidence-based components of therapy for breast, lung, and ovarian carcinomas. In the adjuvant treatment of breast cancer, these drugs are most often used in sequence or in combination with trastuzumab (Herceptin®), doxorubicin and cyclophosphamide [3].

Systemically, docetaxel most commonly causes myelosuppression, neuropathy, myalgias, fatigue, alopecia, diarrhoea, mucosal toxicity and skin and nail changes [1]. Rare ophthalmic adverse effects include epiphora [4,5], canalicular stenosis [6], conjunctivitis [7] and cystic maculopathy [8,9]. Although docetaxel has not previously been reported to cause optic neuropathy, paclitaxel has been implicated in causing transient visual disturbances which mainly occur during drug infusion and is thought to be due to vascular dysregulation in retinal and optic nerve pathways [10,11].

We describe the first reported case of toxic optic neuropathy caused by docetaxel chemotherapy and discuss its significance in the context of previous hypothesized mechanisms of optic nerve damage caused by taxanes.

## Case presentation

A 53-year-old female was admitted to hospital after presenting with a 2-week history of gradually worsening visual loss in the left eye. She described visual loss which mainly involved her left inferior visual field. She reported that her symptoms had started 2 days after infusion of her first cycle of docetaxel/trastuzumab and had gradually worsened since that time. She did not report any visual disturbance at the time of infusion. She reported no other flashes, floaters, or foreign body sensation. She had no complaints of pain, headache, red eyes, irritation, lacrimation or photophobia neither jaw claudication, myalgias, arthalgias nor weight loss. Furthermore, she denied fevers or any other systemic symptoms.

She was hyperopic but otherwise had no other past ophthalmic history. She was an active smoker with a 20-pack year history (currently 10/day) who consumed on average 30 grams of alcohol per day. She otherwise had a well-balanced diet and had no known exposures to any environmental toxins.

She had a past medical history of well-controlled hypertension, gastro-oesphageal reflux disease and hepatic focal nodular hyperplasia (FNH). She had previously had a hysterectomy for fibroids but still had her ovaries in situ.

Her diagnosis of breast cancer occurred 5 months earlier. Initial treatment involved left breast wide local excision and sentinel node biopsy, which was followed by a completion left mastectomy with axillary lymph node sampling. Histology of the operative specimen was consistent with a high risk, multi-focal early stage breast cancer as detailed below:

•Lesion 1- 15 mm × 1.1 mm Grade 3 Invasive Ductal Carcinoma with extensive lymphovascular invasion. ER (oestrogen receptor) 90% positive, PR (progesterone receptor) 20% positive and HER2 positive by SISH (silver in-situ hybridization).

•Lesion 2: 3.5 mm Grade 1 Invasive lobular carcinoma. ER positive, PR positive, HER2 negative

•There was extensive high-grade ductal carcinoma in situ (DCIS) of 120 mm on remaining mastectomy specimen. One out of four axillary lymph nodes was involved with 3.2 mm macro-metastasis.

Staging CT scan showed a 3.8 cm liver lesion straddling Segment IVA and Segment VIII, consistent with her known FNH. There was no evidence of metastatic visceral disease and a bone scan also ruled bony metastasis.

She was planned to receive adjuvant chemotherapy as per Slamon et al. called AC-TH involving 4 cycles of 3 weekly doxorubicin and cyclophosphamide (AC); followed by 4 cycles of 3 weekly concurrent docetaxel and trastuzumab (TH); followed by 3 weekly trastuzumab up to a total of 52 weeks of treatment [12]. Her body surface area (BSA) [13] was 1.83 m2 (height: 169 cm, weight: 71.4 kg) and she had normal baseline renal, hepatic and haemotological function. She was dosed with docetaxel 185 mg (100 mg/m2 BSA) and Herceptin 572 mg (8 mg/kg loading dose) [12].

She tolerated her initial 4 cycles of doxorubicin/cyclophosphamide well, but presented with visual disturbance shortly after her first cycle docetaxel and trastuzumab. At presentation, her regular medications included atenolol, telmisartan, hydrochlorothiazide, pantoprazole, fish oil, multivitamin, vitamin D and glucosamine. She was allergic to codeine.

**Figure 1:**
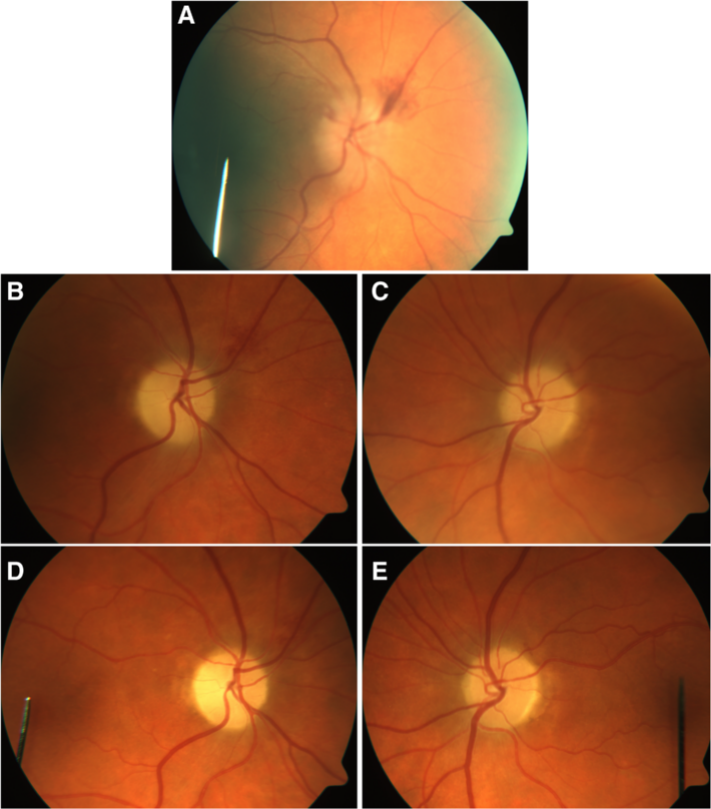
**Optic disc photographs. A**- Right Optic Disc on Presentation, unfortunately the Left Optic Disc could not be photographed adequately. **B**- Right Optic Disc 1 Month later with swelling and hemorrhage resolving. **C**- Left Optic Disc 1 Month later with swelling resolving. **D**- Right Optic Disc 3 Months later. **E**- Left Optic Disc 3 Months later.

Systemic examination on presentation was normal with no focal neurology detected. Vital signs were stable with no hypertension. On ophthalmic examination, her best-corrected visual acuity was 6/7.5 in the right eye and 6/9 in the left eye. A left relative afferent pupillary defect was present. Intraocular pressures were 17 in the right eye and 19 in the left eye. Ishihara plate testing showed 15/15 with a fast response in the right eye and 11/15 with a slow response in the left eye. Red saturation testing showed a 40% reduction in the left eye. Extraocular movements were full and not painful. Temporal arteries were pulsatile and non-tender and there was no carotid bruit. On slit lamp examination, anterior segments were quiet apart from mild nuclear sclerotic cataracts bilaterally. Fundus examination showed swollen optic nerve heads with associated disc haemorrhages bilaterally (Figure 1A). The maculae, retinal vasculature and retinal peripheries were otherwise normal in appearance bilaterally.

**Figure 2:**
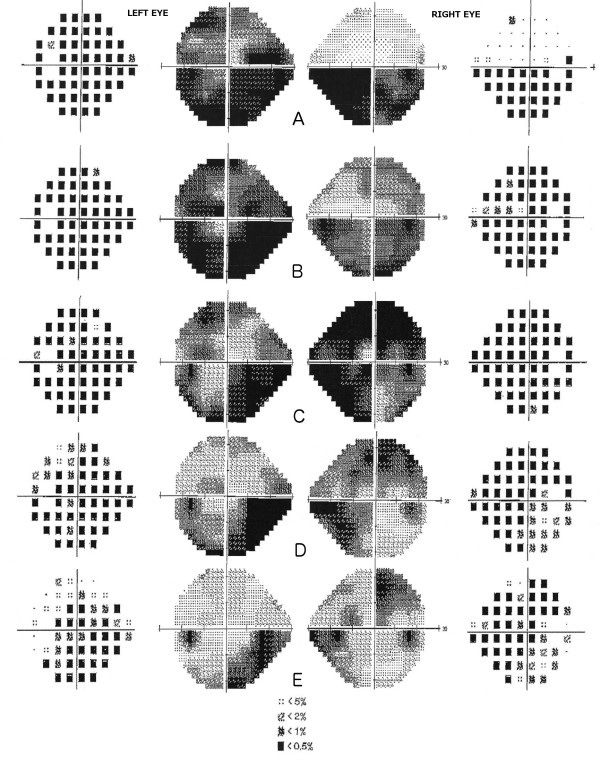
**Evolution of visual field testing (24-2) over the course of docetaxel toxic optic neuropathy (Total Deviation and Grayscale). A**- On Presentation, **B**- 5 days after presentation, **C**- 3 weeks after presentation, **D**- 5 weeks after presentation, **E**- 13 weeks after presentation.

In terms of initial investigations, visual field testing showed inferior field loss in the right eye and widespread field loss in the left eye (Figure 2A). MRI/MRV Head and Orbits with gadolinium contrast was unremarkable with no signs of optic nerve pathology, demyelination, sinus thrombosis, space occupying brain metastasis or leptomeningeal disease. Routine blood work showed a normal full blood count, electrolytes, renal function and liver function. Inflammatory markers were not raised.

After extensive discussion and collaboration between the oncology, neurology and ophthalmology teams, the working diagnosis of a bilateral toxic optic neuropathy was thought to be most likely with docetaxel the likely causative agent rather than trastuzumab. The patient was admitted under the oncology team and treated with high dose steroids: initially dexamethasone 8 milligrams twice daily orally for 24 hrs, followed by intravenous pulse methylprednisolone 1 gram once daily for 3 days on neurology advice.

Over the next 36 hours more extensive investigations were undertaken. Further blood work including ANA, dsDNA, Rheumatoid factor, ANCA, ACE, antiphospholipid antibodies, and anti-neuronal antibodies (anti-HU/Ri/Yo/PCA-2) were all negative. Quantiferon Gold, Toxoplasma and syphilis serology and CMV/EBV IgM were also negative. Serum levels of vitamins potentially causing a nutritional optic neuropathy were normal as well. Chest X-ray was unremarkable. Lumbar puncture showed an opening pressure of 11 cm of H_2_O and CSF analysis showed no leucocytes or blood, and protein and glucose levels within normal limits. Cryptococcal serology and HSV PCR were negative on the CSF sample. Transthoracic echocardiography and carotid ultrasound were both normal. CT Chest/Abdomen/Pelvis was unremarkable. Unfortunately ophthalmic electrophysiological testing was not possible in this patient.

Over the first 36 hours of the admission, ophthalmic examination revealed gradual worsening in best-corrected visual acuity to 6/30 in the right eye and 6/120 in the left eye, although the patient’s vision was seen to fluctuate over this period. Serial visual field testing also showed progression of visual field defects bilaterally despite a stable optic nerve head appearance on examination (Figure 2B). On the 4^th^ day of admission, after 3 doses of methylprednisolone, the patient reported slight improved in central vision bilaterally with best-corrected visual acuity of 6/9 in the right eye and 6/9 in the left eye. Visual field testing at this time was stable in both eyes (Figure 2C). The patient was then discharged from hospital on 70 mg of oral prednisone daily which was to be tapered down over 2 months.

Subsequent review over the next 3 months showed stabilisation of visual acuity, resolution of optic nerve head swelling and haemorrhage (Figure1 B-E) and resolution of visual field defects bilaterally (Figure 2D-E). Docetaxel chemotherapy was discontinued but the patient has since completed 8 further cycles of trastuzumab therapy without any complication. Steroids were successfully ceased with no relapse of symptoms.

After biochemical assessment of the pituitary-gonadal axis confirmed that the patient was postmenopausal, adjuvant endocrine therapy with an aromatase inhibitor – letrozole (1-letrozole) was safely commenced with no deterioration in vision. Three months after discharge, best-corrected visual acuity was 6/9 bilaterally. Trastuzumab therapy is planned to continue for 1 year in total in addition to 5 years of letrozole therapy as per international guidelines [14]. The patient was strongly encouraged to quit smoking.

## Conclusion

Wang and Sadun recently proposed 5 postulates required for establishing a causal link between a toxic agent and an optic neuropathy [15]. These include having a strong biological basis for the optic neuropathy, an association demonstrated on a dose-response curve, long duration of toxin exposure as a risk factor, recovery on discontinuation of the toxin and predominant bilaterally of the neuropathy. We propose that our patient developed a docetaxel-induced toxic optic neuropathy due to the strong temporal relationship between docetaxel administration and subsequent development of bilateral visual loss and visual field defects. This then correlates with bilateral visual recovery after docetaxel therapy discontinuation and treatment with corticosteroids. The extensive investigations our patient underwent also discount other causes for her optic neuropathy. In our case, trastuzumab was continued due to its indispensable role on the patient’s malignancy related prognosis. To our knowledge, there has only been 1 described case of trastuzumab induced ocular toxicity and that case involved macular ischaemia [16]. In addition, trastuzumab was discounted as the causative agent because further trastuzumab cycles have not reproduced optic neuropathy.

Because docetaxel has not previously been reported to cause optic neuropathy, the scientific rationale for the causative mechanism of this condition is difficult to explain from a single reported case. However, we hypothesize that this toxic neuropathy is most likely caused by an underlying ischemic or neurotoxic insult to the optic nerve retinal ganglion axons. Although there is limited literature linking docetaxel to optic nerve dysfunction, the other major taxane, paclitaxel, has been previously reported to cause optic nerve dysfunction on both electroretinography and visual-evoked potential testing [17]. These optic nerve changes were reported in patients who experienced scintillation scotoma and photopsias mainly during paclitaxel infusion [18-20]. These symptoms were usually transient but some patients did have prolonged visual loss. Other studies have linked this symptomatology to electrophysiological changes in the retina and optic nerve [10,17,21] with Scaioli et al. [17] proposing that these changes could be caused by vascular dysregulation and potentially vasospasm in retinal and optic nerve vasculature. This vascular hypothesis could be supported by the clinical appearance of optic nerve head swelling/haemorrhages in our patient that was consistent with a non-arteritic acute ischemic optic neuropathy. Interestingly, this appearance parallels the optic neuropathy associated with interferon alpha therapy which is also thought to be caused by ischemia [22].

In contrast to a potential vascular mechanism, other authors have hypothesized that taxane-induced optic nerve dysfunction could also potentially be due to neurotoxicity of the optic nerve [19]. Gianni et al. [19] suggest that optic nerve dysfunction could be caused by a similar mechanism to that which causes the peripheral neuropathy induced by both docetaxel and paclitaxel. Although the mechanism of this neurotoxicity has not yet been fully elucidated, it is known that taxanes promote the aggregation of intracellular microtubules in axons leading to dysfunction of axonal transport and thus leading to distal sensory and motor axonal neuropathy. It is well documented that taxanes easily penetrate the blood-brain barrier, therefore it seems logical that this same mechanism could also occur in the central nervous system leading to direct axonal injury and neuropathy [23,24].

Whether the pathogenesis of this condition is due to optic nerve head ischemia, neurotoxicity or another mechanism remains to be demonstrated. Whilst it is conceivable that docetaxel therapy was unrelated to our patient’s optic neuropathy, we believe it is difficult to discount our hypothesis due to the temporal relationship between docetaxel infusion and symptom onset, subsequent resolution and the lack of other underlying risk factors, medications or evidence of other cause. Our case demonstrates that discontinuation of docetaxel therapy and treatment with corticosteroids to address any associated optic nerve inflammation is efficacious in halting the progression of a possible docetaxel-induced optic neuropathy and can result in visual recovery over several months. We recommend that all patients who experience visual disturbance during docetaxel chemotherapy should be examined by an ophthalmologist so that potentially blinding long term sequelae are avoided.

### Requesting consent statement

Written informed consent was obtained from the patient for publication of this case report and any accompanying images. A copy of this written consent is available for review by the Editor of this Journal.

## Competing interests

The authors declare that they have no competing interests.

## Author’s contributions

KRB, WX, NW, ON and JF initially identified the case. TM and WX conducted the literature review and reviewed the patient’s medical records. TM, WX, NO and NW drafted the manuscript. All parties approved the final manuscript.

## Pre-publication history

The pre-publication history for this paper can be accessed here:

http://www.biomedcentral.com/1471-2415/14/18/prepub
